# Rapid, automated, and reliable antimicrobial susceptibility test from positive blood culture by CAST‐R

**DOI:** 10.1002/mlf2.12019

**Published:** 2022-04-18

**Authors:** Pengfei Zhu, Lihui Ren, Ying Zhu, Jing Dai, Huijie Liu, Yuli Mao, Yuandong Li, Yuehui He, Xiaoshan Zheng, Rongze Chen, Xiaoting Fu, Lili Zhang, Lijun Sun, Yuanqi Zhu, Yuetong Ji, Bo Ma, Yingchun Xu, Jian Xu, Qiwen Yang

**Affiliations:** ^1^ Single‐Cell Center, CAS Key Laboratory of Biofuels, Shandong Key Laboratory of Energy Genetics and Shandong Energy Institute, Qingdao Institute of Bioenergy and Bioprocess Technology Chinese Academy of Sciences Qingdao China; ^2^ University of Chinese Academy of Sciences Beijing China; ^3^ College of Information Science & Engineering Ocean University of China Qingdao China; ^4^ Department of Clinical Laboratory, Peking Union Medical College Hospital, Peking Union Medical College Chinese Academy of Medical Sciences Beijing China; ^5^ Graduate School, Peking Union Medical College Chinese Academy of Medical Sciences Beijing China; ^6^ Department of Clinical Laboratory, Affiliated Hospital of Qingdao University Qingdao University Qingdao China; ^7^ Qingdao Single‐Cell Biotechnology, Co., Ltd. Qingdao China; ^8^ The Bioland Laboratory Guangzhou China

**Keywords:** antimicrobial susceptibility test, laboratory automation, Raman, sepsis, tigecycline

## Abstract

Antimicrobial susceptibility tests (ASTs) are pivotal in combating multidrug resistant pathogens, yet they can be time‐consuming, labor‐intensive, and unstable. Using the AST of tigecycline for sepsis as the main model, here we establish an automated system of Clinical Antimicrobials Susceptibility Test Ramanometry (CAST‐R), based on D_2_O‐probed Raman microspectroscopy. Featuring a liquid robot for sample pretreatment and a machine learning‐based control scheme for data acquisition and quality control, the 3‐h, automated CAST‐R process accelerates AST by >10‐fold, processes 96 paralleled antibiotic‐exposure reactions, and produces high‐quality Raman spectra. The Expedited Minimal Inhibitory Concentration via Metabolic Activity is proposed as a quantitative and broadly applicable parameter for metabolism‐based AST, which shows 99% essential agreement and 93% categorical agreement with the broth microdilution method (BMD) when tested on 100 *Acinetobacter baumannii* isolates. Further tests on 26 clinically positive blood samples for eight antimicrobials, including tigecycline, meropenem, ceftazidime, ampicillin/sulbactam, oxacillin, clindamycin, vancomycin, and levofloxacin reveal 93% categorical agreement with BMD‐based results. The automation, speed, reliability, and general applicability of CAST‐R suggest its potential utility for guiding the clinical administration of antimicrobials.

## Impact statement

Rapid and reliable antimicrobial susceptibility tests (ASTs) are pivotal to effective, safe, and environmentally responsible treatment of acute infections such as sepsis. For example, conventional ASTs for tigecycline, a last‐resort antimicrobial treating blood infections, have been slow, tedious, and even plagued by inaccuracy, due to the drug's sensitivity to the dissolved oxygen in broth. Here employing tigecycline as the main model, we establish an automated system of Clinical Antimicrobials Susceptibility Test Ramanometry (CAST‐R), based on D_2_O‐probed Raman microspectroscopy. Results from *Acinetobacter baumannii* isolates as well as clinically positive blood samples show that the 3‐h CAST‐R process achieves excellent accuracy yet accelerates AST by >10‐fold.

## INTRODUCTION

For effective, safe, and environmentally responsible treatment of multidrug‐resistance (MDR) infections, rapid and reliable antimicrobial susceptibility tests (ASTs) are pivotal^1^. For example, for sepsis that carries a 20%–40% mortality rate^2^, resistance to last‐resort antimicrobials including tigecycline^3^ has emerged in many important blood pathogens, such as *Acinetobacter baumannii* ^4^, yet each hour's delay in turn‐around time (TAT) of AST elevates mortality rate by 7.6%^5^. Therefore, rapid, reliable, and clinically deployable ASTs are of particular urgency in treating sepsis^3^.

At present, the gold standard method for *in vitro* AST of tigecycline is culture‐based broth microdilution (BMD^6^); however, its TAT from positive blood culture (PBC) to AST results is usually 36–48 h (for tigecycline and other antimicrobials^7^). Moreover, BMD is exceedingly tedious as fastidious conditions are required for culture and incubation (so as to avoid deactivation of tigecycline by the dissolved oxygen in broth^8^). Furthermore, commercial AST systems, such as VITEK 2 and E‐test, often overestimate the minimal inhibitory concentration (MIC) of tigecycline or even report false resistance^9^, ^10^.

We and others recently established D_2_O‐probed single‐cell Raman Microspectroscopy (D_2_O Ramanometry) as a culture‐independent, metabolism‐based AST^11^, ^12^, ^13^, ^14^, ^15^, ^16^. Each single‐cell Raman spectrum (SCRS), composed of thousands of Raman peaks that individually or collectively represent the resonance frequency of chemical bonds from intracellular metabolites, is a proxy of the metabolic state of the cell. When D_2_O is fed to a live cell, incorporation of deuterium into the newly synthesized macromolecules would produce, in an otherwise silent region (2040–2300 cm^−1^), the “C−D band,” whose intensity indicates the amount of D_2_O consumption^17^. The C–D ratio (i.e., CDR), defined as a percentage of the integrated spectral intensity of the C–D band (2040–2300 cm^−1^) to sum of the C–D band and the “C–H band” (2800–3100 cm^−1^), can quantitatively model metabolic activity of the cell. Thus, ΔCD/(CD + CH) (i.e., ∆C–D‐ratio or ∆CDR), the temporal change of CDR after drug exposure relative to the average CDR in the absence of drug, was proposed to model the degree of cellular metabolic inhibition by drug exposure^11^. Correspondingly, we proposed the concept of Minimal Inhibitory Concentration via Metabolic Activity (MIC‐MA) as a quantitative measure for AST^11^. Such measurement of cellular D_2_O intake rate (i.e., metabolic vitality) instead of the propagation of cells as a measurement of drug susceptibility greatly reduces the TAT of AST results^11^, ^12^, ^13^, ^14^, ^15^, ^16^.

However, whether AST of tigecycline can be tackled by D_2_O Ramanometry is not clear, particularly in light of the distinct mechanism between D_2_O Ramanometry and BMD: the former quantifies tigecycline‐induced change in cellular metabolic activity, while the latter tracks alteration of cellular growth and propagation^11^. More importantly, clinical application for bloodstream infections necessitates rapid, high throughput, ease in use (i.e., automation), and reliability of results^1^, yet these have not been demonstrated for the present designs of D_2_O Ramanometry‐based ASTs^11^, ^12^, ^13^, ^14^, ^15^, ^16^. Specifically, pivotal to the clinical deployment of D_2_O Ramanometry are: (i) automation in sample preparation that allows paralleled processing of multiple clinical samples, (ii) intelligent acquisition and quality control of Raman microspectroscopy that supports the fully automated acquisition of high‐quality Raman spectra, (iii) a reliable algorithm to quantitatively define drug susceptibility, and (iv) thorough validation of result reliability on clinical samples and then standardization of the whole process at the clinical setting.

**Figure 1:**
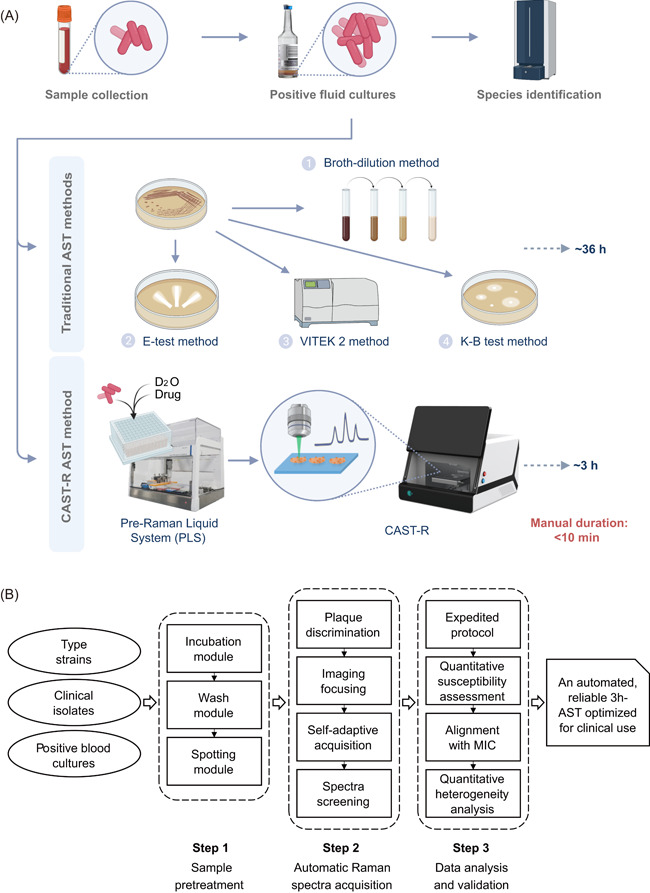
A global overview of the automated workflow of CAST‐R. (A) Distinction between the CAST‐R workflow and the traditional, culture‐based AST workflows, which is illustrated using the AST of clinical blood samples as an example. (B) Key challenges and solutions for the development of CAST‐R. AST, antimicrobial susceptibility test; CAST‐R, Clinical Antimicrobials Susceptibility Test Ramanometry.

To tackle these challenges, we employed AST of tigecycline for sepsis as a main model and established the automated Clinical Antimicrobials Susceptibility Test Ramanometry (CAST‐R; Figure 1A). The 3‐h, automated CAST‐R procedure reduces time‐to‐result from PBC by >10‐fold, processes 96 paralleled antibiotic‐exposure reactions and produces Raman spectra with quality equivalent to those manually acquired. The automation, speed, reliability, and broad applicability suggest CAST‐R as a clinically valuable AST for bloodstream infections.

## RESULTS

### An overview of the automated CAST‐R system

CAST‐R features an automated AST workflow that starts from PBC (Figure 1A,B). Before CAST‐R, blood samples are collected and enriched in commercial blood culture medium for 12–24 h, then those blood samples reported as positive (i.e., PBC) would enter the CAST‐R workflow for AST (with another aliquot undergoing MALDI‐TOF for pathogen identification, in parallel with CAST‐R; Figure 1B). First, the PBC is pretreated via the Pre‐Raman Liquid System (PLS), an automated module of the CAST‐R instrument for D_2_O incubation, cell washing, and spotting onto detection chips. Second, Raman spectra of each sample on chips are automatically acquired via the Raman microspectrometer module of CAST‐R by intelligent spot focusing and spectra filtering. Finally, the antimicrobial susceptibility of clinical samples is modeled via expedited MIC‐MA (eMIC‐MA). The whole process yields AST results ~ 3 h from PBC. In contrast, culture‐based AST methods, such as VITEK 2, E‐test, and BMD usually take >48 h starting from PBC.

### Automated sample preparation for CAST‐R from clinically PBC

As the first step of a Raman‐based AST workflow, sample preparation usually consists of several manual, very tedious operations: (i) introduction of antimicrobial sets and addition of D_2_O, which can be error‐prone; (ii) multiple rounds of thorough cell washing to avoid interference by background signal, which can cost at least 20 min; (iii) spotting of postdrug‐exposure samples on a CaF_2_ slide so that cells are precisely concentrated as a tiny plaque, which is particularly important for low‐biomass samples (<10^4^ CFU/ml).

To automate the sample preparation process, an automated liquid‐handling work station called PLS was introduced that consists of the incubation module, the sample‐wash module, and the spotting module. Specifically, an 8‐channel‐pipettor robot combined with preprepared 96‐well antimicrobial assay was employed for sample transfer and incubation. A vacuum‐based 96‐well filter plate was used for automated sample wash. Then a hydrophobic quartz chip was adopted for automatic spotting to elevate the cell density of the spotted sample, on which Raman spectra over multiple samples were acquired automatically. By handling 96 reactions simultaneously, PLS reduces the total time burden of manual operations (i.e., loading reagents, tips, and samples onto the platform) to <5 min, in contrast to the manual procedure that takes >3 h^11^, ^12^, ^13^, ^14^, ^15^, ^16^.

### Automated acquisition of high‐quality Raman spectra in CAST‐R

Although commercial Raman spectrometers can include microscopy modules that feature auto‐focusing and spectrum acquisition, manual operations are still required and the quality of spectra is heavily dependent on user experiences. This is not suitable for a clinical laboratory where automatic acquisition of many clinical samples in a parallel and intelligent quality screening of spectra are required. Thus, we propose a fully automated high‐throughput solution, which includes plaque discrimination from sample chip, image auto‐focusing, spectra auto‐collecting, and automated quality assessment and screening functions.

**Figure 2:**
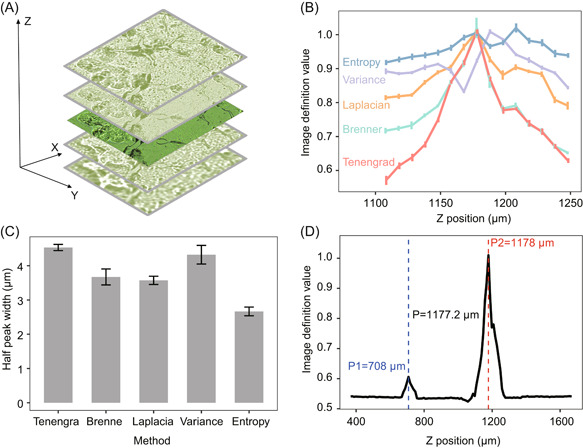
Automated and intelligent spectrum collection in CAST‐R. (A) Auto‐focusing process based on cell image. (B) Definition evaluations of the focusing and defocusing images with different methods by computing the definition values of captured images. (C) The full widths at half maximum (FWHM) of different image definition evaluation methods. (D) The effect of our proposed search method Tenengrad. P1 is the local maximum image definition on the position of 708 µm, and P2 is the optimal position identified by our search method. P2 is gradually closing to the actual best focusing position (P).

First, to quickly locate the spotted plaque on the sample chip, a plaque discrimination method was developed. Based on the brightness value of the image, the area of plaque was then defined. Second, pinpointing the accurate imaging focus (Z position) of plaque is the prerequisite for acquiring high‐quality spectra, thus an auto‐focusing algorithm was implemented to find the optimal imaging focus (Figure 2A). Image definition value was introduced as a measurement for the quality of images, and performance was compared among five search methods (Entropy, Variance, Laplacian, Brenner, and Tenengrad; Figure 2B), to find the suitable imaging focus that produces the highest image quality^18^, ^19^. Tenengrad offers the best performance, due to the highest half peak width (Figure 2B,C) and the ability to discriminate against false peaks (i.e., false focuses such as P1 in Figure 2D). Third, after pinpointing optimal focus, CAST‐R automatically initiates spectrum acquisition and adaptively adjusts detection parameters, such as “laser power,” “exposure time,” and “acquisition interval” (according to spectrum quality) to obtain high‐quality spectra.

Finally, we developed an intelligent filter, based on convolutional neural networks (CNN), for automated quality screening of the Raman signal. Specifically, the signal quality can vary greatly due to the presence of diverse cellular states after drug exposure; moreover, saturated spectra (i.e., signals from the cell burned by the laser, whose intensity is unusually high and does not match the cell's intrinsic signal) and spectra of low signal‐to‐noise ratio (SNR < 30) can both significantly interfere with AST assessment. To evaluate spectrum quality properly and efficiently, a database of high‐quality reference SCRS was created for spectra quality assessment of test samples, via SNR and the overall pattern of a spectrum that depicts a cell. For the database, over 10,000 high‐quality SCRS (SNR ≥ 30) from 13 species (15 strains) of the most frequently encountered pathogens in blood infections were collected (Table S1). Then the maximum probability of the input spectra's species assignment was calculated by CNN and compared with a threshold to determine the quality of input spectra.

To evaluate the performance of the spectra‐quality filter in automated screening, three simulated datasets of 100 spectra were each produced via mixing spectra of high‐quality (SNR ≥ 30) and low‐quality (SNR < 30, saturated spectra, or spectra with outlier spikes) at three ratios: 9:1 (“Dataset A”), 7:3 (“Dataset B”), and 5:5 (“Dataset C”). For each of the data sets, the performance of automated screening (and manual screening by three experts) was evaluated by comparison to the ground truth (i.e., the “Reference”), via parameters for spectra quality and throughput. For sensitivity, specificity, SNR, and C–D ratio, both automatic and manual methods fully and accurately reconstruct the ground truth, with no significant difference in performance detected (Figure S1A–C). However, the automatic method represents on average 66‐fold acceleration in data screening (i.e., 59‐, 64‐, and 74‐folds for Dataset A, B, and C, respectively; Figure S1D). Therefore, our automated data collection and screening procedure can replace the tedious manual assessment of spectra quality, which is crucial in clinical practice.

### Quantitating tigecycline susceptibility of *A. baumannii* via the “eMIC‐MA”

To develop a tigecycline AST via the automated CAST‐R, *A. baumannii* was chosen as a model, as this bacterium is among the most frequently encountered pathogens^4^, showing a higher resistance rate to tigecycline than other MDR pathogens^9^. An optimized protocol was established as a standard, which includes: (i) using fresh Cation‐Adjusted Mueller–Hinton Broth (CAMHB) as incubation medium (based on CLSI protocol^20^); (ii) incubating samples for 1 h under series dilutions of tigecycline; (iii) adding 30% D_2_O after the initial tigecycline exposure and then incubating for another 1 h before Raman detection; (iv) adopting Epidemiological Cut‐off (ECOFF) as the MIC breakpoint.

To derive a golden reference for interpreting AST results, a suitable MIC breakpoint is usually required. However, in the CLSI and FDA criteria for AST interpretation, there are no MIC breakpoints of tigecycline for *Acinetobacter* spp. at present. In EUCAST, based on the international distribution pattern of MIC of tigecycline for *Acinetobacter* spp. (Figure S2), the ECOFF has been set as 0.5 mg/l^21^. Therefore, for validating CAST‐R‐derived AST results, the MIC breakpoints of tigecycline for *Acinetobacter* spp. are designated as ≤0.5 mg/l for “susceptible organisms” (S^21^), and as ≥1 mg/l for “nonsusceptible organisms” (NS).

**Figure 3:**
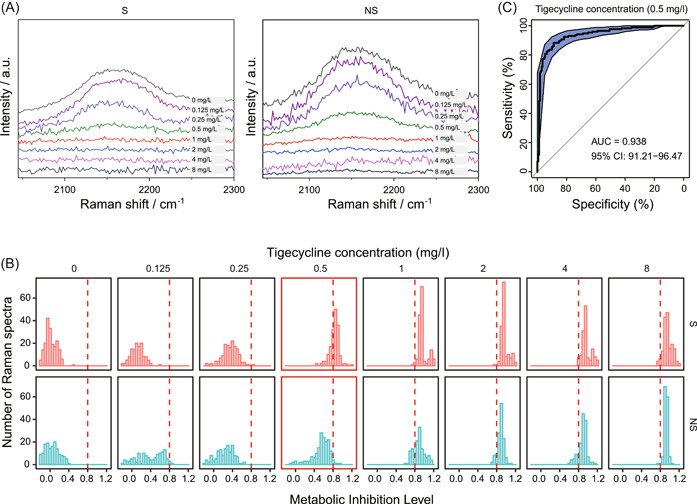
Quantitating tigecycline susceptibility of *A. baumannii* via eMIC‐MA using CAST‐R. (A) Comparison of the spectral change of C–D peak for the S and NS strains under different doses of tigecycline. (B) The counts of Raman spectra of the S and NS strains as a function of the metabolic inhibition level (MIL) of various doses of tigecycline after 2 h incubation with tigecycline. The red dashed lines (0.8) are the criteria to distinguish the S and NS strains. (C) The ROC curve for discriminating the S and NS strains under 0.5 mg/l of tigecycline. AUC, area under the curve; NS, nonsusceptible organisms; S, susceptible organisms; ROC, receiver operating characteristic.

To test the validity of these designations, the S strain of *A. baumannii* A29 (MIC = 0.5 mg/l) and the NS strain of *A. baumannii* A30 (MIC = 1 mg/l) were probed under series tigecycline doses via CAST‐R. Under 0.5 mg/l tigecycline, for A29, no C–D peaks (2040–2300 cm^−1^) were detected. In contrast, A30 showed an apparent C–D peak under tigecycline (Figure 3A). Therefore, the designated breakpoints of tigecycline are suitable for distinguishing S and NS *A. baumannii* strains via CAST‐R.

To quantify antimicrobial susceptibility via SCRS, we have previously proposed MIC‐MA based on ∆CDR, the metabolic activity change after drug exposure^11^. Specifically, when we first proposed D_2_O Ramanometry as a rapid AST, MIC‐MA is defined as the minimal dose under which the median ∆CDR at 8 h of drug exposure is ≤0 and the standard deviation (SD) of the ∆CDR among individual cells is ≤0.005^11^. However, as SCRS‐based AST can be much faster than 8 h (e.g., 15–30 min for discriminating sodium fluoride susceptibility for *Streptococcus mutans* ^11^), we further propose the “Expedited MIC‐MA”, as defined below.

First, the “Metabolic Inhibition Level” (MIL) was introduced to quantify the extent of cellular metabolic suppression after antimicrobial treatment. To normalize against potential variation due to change of strains, starting state of cells, or instruments, MIL was derived as: $\mathrm{MIL}=\frac{{\mathrm{CDR}}_{\mathrm{control}}-{\mathrm{CDR}}_{\mathrm{treated}}}{{\mathrm{CDR}}_{\mathrm{control}}-{\mathrm{CDR}}_{0\;h}}$. The mean MIL of groups under different antimicrobial doses would range from 0 to 1, with a higher MIL value indicating a higher level of metabolic inhibition. MIL is advantageous versus ∆C–D ratio, as the degree of cellular deuterium utilization can vary greatly among different species and strains^13^.

Second, the proper cutoff value of MIL was determined for deriving eMIC‐MA. Specifically, the MIL histograms for the S and NS strains were aligned, which reveals that the patterns are largely consistent with nearly all tested levels of tigecycline, except under 0.5 mg/l when the two patterns become highly distinct (Figure 3B). Moreover, under 0.5 mg/l tigecycline, among the MIL values that span from 0 to 1, a cutoff value of 0.8 was found to maximally distinguish between the S and NS strains (Figure 3B). Based on these settings, the tigecycline susceptibility of S and NS strains of *A. baumannii* is distinguished with very high accuracy (area under the curve = 0.938; Figure 3C).

Finally, eMIC‐MA was defined as the minimal dose under which mean MIL after 2 h of tigecycline exposure is ≥0.8 (i.e., the cutoff value of MIL as defined above). Therefore, eMIC‐MAs of these two *A. baumannii* strains are 0.5 and 1 mg/l, respectively, which are identical to the MIC reported by BMD. Altogether, via the tigecycline preincubation, adaptation of EUCAST breakpoints, and designation of eMIC‐MA, CAST‐R is able to determine tigecycline AST for *A. baumannii* within 3 h.

### Tests on 100 clinical *A. baumannii* isolates validate the accuracy and reliability of tigecycline AST via eMIC‐MA using CAST‐R

**Figure 4:**
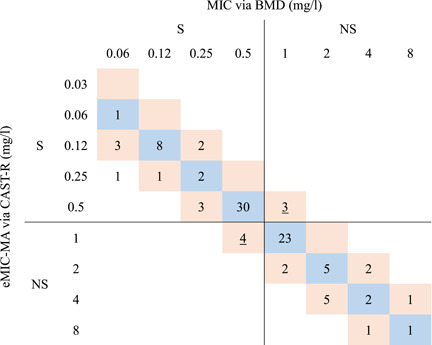
Comparison of MICs and eMIC‐MAs of the clinical *A. baumannii* isolates (*n* = 100). The tigecycline susceptibility properties derived via eMIC‐MA are in excellent agreement with those based on MIC, showing 99% EA (99/100, marked with blue and red) and 93% CA (93/100; seven discrepant isolates marked with underlines).

To test the accuracy of CAST‐R for clinical isolates that span various MICs for tigecycline, we further analyzed 100 clinical isolates of *A. baumannii* (Figure 4). Their MICs, measured via BMD based on the CLSI protocol, range from 0.0625 to 8 mg/l and their distribution pattern is similar to the EUCAST collections (Figure S2). These overnight cultured isolates were suspended to 5 × 10^5^ CFU/ml and then processed to validate our 3 h CAST‐R workflow. Each isolate was treated by at least seven dilutions of tigecycline with the tigecycline‐free group as control. The mean MIL under 0.5 mg/l of each isolate was used to discriminate tigecycline susceptibility (cutoff value set as 0.8).

The 100 isolates can be classified into two classes based on MIC (Figures 4 and S3). For most S strains (MIC ≤ 0.5 mg/l) except strain A32, A53, A73, and XHAB13, their D_2_O‐intake activities are inhibited under 0.5 mg/l tigecycline (mean MIL_0.5 mg/l_ ≥ 0.8), thus their eMIC‐MA are no more than 0.5 mg/l. As for most NS strains (MIC ≥ 1 mg/l), their mean MIL_0.5 mg/l_ are below 0.8 under 0.5 mg/l tigecycline, therefore their eMIC‐MA are no lower than 1 mg/l (except strain A15, A60 and A63, whose eMIC‐MA are 0.5 mg/l). Notably, these eMIC‐MA‐based tigecycline AST results are highly consistent with the MIC‐based diagnosis, with a categorical agreement (CA) of 93% (93/100; Figures 4 and S3).

The results support the capability of eMIC‐MA for rapid and reliable classification of tigecycline susceptibility, based solely on a single MIC breakpoint of 0.5 mg/l. However, in clinical practice, the MIC breakpoint for a given microbe‐drug pair can change significantly, due to variation in sample type^20^ or revisions of CLSI recommendations, thus resulting in misinterpretation of AST results^22^. Therefore, a broadly applicable parameter that can quantitatively measure antimicrobial susceptibility (not just classification) is highly desirable. To assess whether eMIC‐MA can meet this requirement, for each of the 100 *A. baumannii* strains, the eMIC‐MA of tigecycline was compared to its corresponding MIC. Notably, nearly all eMIC‐MA of the 100 isolates are identical to or within ±1‐fold dilution of the MIC reported by the BMD method (Figure 4), with the only exception being XHAB15, which is within a twofold dilution difference. This performance represents an essential agreement (EA) of 99% (99/100), supporting eMIC‐MA as a broadly applicable parameter for quantitative measurement of antimicrobial susceptibility.

### Rapid and accurate tigecycline susceptibility test directly from spiked PBC by CAST‐R

To validate its speed and reliability, we next tested CAST‐R for infected blood samples, which were constructed by spiking clinical *A. baumannii* isolates into fresh blood bottles and incubated until the cultures reported positive. The 14 selected strains are clinically significant and exhibit a wide range of susceptibility levels to tigecycline, with MICs ranging from 0.125 to 8 mg/l (Table S2). For CAST‐R, the PBC was incubated with ammonium‐chloride‐potassium (ACK) lysis buffer to lyse blood cells and then centrifuged to remove cell debris, followed by exposure to 0–8 mg/l tigecycline and then D_2_O incubation. In parallel, the BMD method was employed for MIC‐based AST to validate the eMIC‐MA based diagnosis via CAST‐R. Both BMD and CAST‐R methods were repeated in triplicate for all samples. From delivery of PBC to obtaining AST results, the full CAST‐R procedure takes ~3 h, in contrast to the ~36 h BMD procedure, representing >10‐fold acceleration.

**Figure 5:**
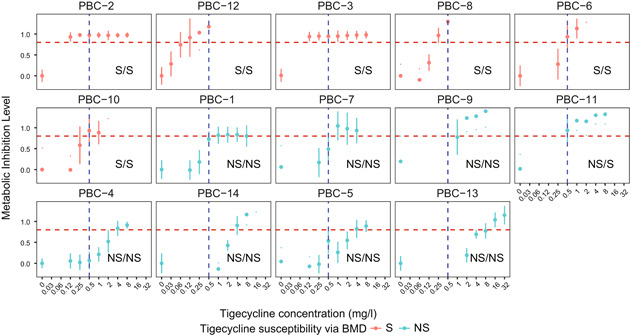
AST of spiked positive blood cultures of *Acinetobacter baumannii* via CAST‐R. The tigecycline‐dose‐dependent change of metabolic inhibition level (MIL), for selected positive blood cultures  (PBCs). The tigecycline concentrations tested range from 0 to 8 mg/l. The red dashed line (0.8) is the cutoff to determine eMIC‐MA. At the bottom right of each panel, tigecycline susceptibility results via either BMD (left) or CAST‐R (right) are provided. The analyses are in triplicates for all samples. eMIC‐MA, Expedited Minimal Inhibitory Concentration via Metabolic Activity.

According to the aforementioned MIL cutoff (0.8), the eMIC‐MA of PBC‐1 to PBC‐14 were calculated as 0.125–16 mg/l. Therefore, seven of the samples were classified as S, while the others as NS (Figure 5). This diagnosis represents a CA of 92.8% with the MIC‐based diagnosis via BMD (13/14; Table S2), which validates the speed and reliability of CAST‐R based tigecycline susceptibility test directly from spiked PBC.

### Tests of eight antibiotics on 26 PBCs demonstrate the general applicability of CAST‐R

To further validate the general applicability of the integrated pipeline of CAST‐R, we next tested CAST‐R performance in actual clinically PBC over a wider range of antibiotics. A total of 26 such PBCs, representing four of the most common clinically encountered pathogens in China^23^: *Escherichia coli* (seven PBCs), *Klebsiella pneumonia* (four PBCs), *Staphylococcus aureus* (six PBCs), and *A. baumannii* (nine PBCs) were enrolled after MALDI‐TOF‐based species identification. Eight antimicrobials, including tigecycline, meropenem, ceftazidime, ampicillin/sulbactam, oxacillin, clindamycin, vancomycin, and levofloxacin were then employed for the tests. Based on the level of clinical priority, 86 pathogen‐antimicrobial pairs were selected for the testing. For each of the 86 pairs, ASTs from PBCs were conducted via the 3‐h CAST‐R procedure, with the 36‐h BMD method also performed in parallel for comparison (Tables 1 and S3). For each of the four pathogens, the breakpoints of tigecycline were chosen based on the EUCAST guideline (0.5 mg/l), while those for the other antimicrobials were based on the CLSI standard.

**Table 1:** Validating CAST‐R‐based AST of 26 clinically positive blood cultures via combinations of four types of pathogens and eight kinds of antimicrobials.

		Antimicrobial susceptibility (MIC/eMIC‐MA)							
Positive blood culture	Pathogen	TGC	MEM	CAZ	SAM	OXA	CLI	VAN	LVX
Abau‐1	*Acinetobacter baumannii*	NS/NS	R/R	R/R	R/R	‐	‐	‐	‐
Abau‐2	*A. baumannii*	NS/NS	R/R	‐	‐	‐	‐	‐	‐
Abau‐3	*A. baumannii*	NS/NS	R/R	‐	‐	‐	‐	‐	‐
Abau‐4	*A. baumannii*	NS/NS	R/R	‐	‐	‐	‐	‐	‐
Abau‐5	*A. baumannii*	S/S	I/I	‐	‐	‐	‐	‐	‐
Abau‐6	*A. baumannii*	NS/NS	I/I	‐	‐	‐	‐	‐	‐
Abau‐7	*A. baumannii*	S/NS	R/R	S/R	S/S	‐	‐	‐	‐
Abau‐8	*A. baumannii*	NS/NS	R/R	‐	‐	‐	‐	‐	‐
Abau‐9	*A. baumannii*	NS/NS	R/R	‐	‐	‐	‐	‐	‐
Eco‐1	*Escherichia coli*	S/S	S/S	R/R	R/R	‐	‐	‐	‐
Eco‐2	*E. coli*	S/S	S/S	‐	‐	‐	‐	‐	‐
Eco‐3	*E. coli*	S/S	S/S	‐	‐	‐	‐	‐	‐
Eco‐4	*E. coli*	S/S	S/S	S/R	I/R	‐	‐	‐	‐
Eco‐5	*E. coli*	S/S	S/S	S/S	S/S	‐	‐	‐	‐
Eco‐7	*E. coli*	S/S	S/S	‐	‐	‐	‐	‐	‐
Eco‐8	*E. coli*	S/S	S/S	‐	‐	‐	‐	‐	‐
Kpn‐1	*Klebsiella pneumoniae*	S/S	S/I	S/S	S/S	‐	‐	‐	‐
Kpn‐2	*K. pneumoniae*	S/S	S/S	‐	‐	‐	‐	‐	‐
Kpn‐3	*K. pneumoniae*	S/S	S/S	S/S	R/R	‐	‐	‐	‐
Kpn‐4	*K. pneumoniae*	S/S	S/I	S/S	S/S	‐	‐	‐	‐
Sau‐1	*Staphylococcus aureus*	S/S	‐	‐	‐	S/S	R/R	S/S	S/S
Sau‐3	*S. aureus*	S/S	‐	‐	‐	S/S	S/S	S/S	S/S
Sau‐5	*S. aureus*	S/S	‐	‐	‐	S/S	S/S	S/S	S/S
Sau‐6	*S. aureus*	S/S	‐	‐	‐	S/S	S/S	S/S	S/S
Sau‐7	*S. aureus*	S/S	‐	‐	‐	S/S	S/S	S/S	S/S
Sau‐8	*S. aureus*	S/S	‐	‐	‐	S/S	R/R	S/S	S/S

The antimicrobial susceptibility results measured based on eMIC‐MA (via the CAST‐R system) are consistent with those based on MICs (via the BMD method), with a categorical agreement of 93%. Four of the most frequently encountered pathogens in hospitals in China^23^ included: *E. coli, K. pneumonia, S. aureus*, and *A. baumannii*. Full experimental details are provided in Table S3. CAZ, ceftazidime; CLI, clindamycin; LVX, levofloxacin; MEM, meropenem; OXA, oxacillin; SAM, ampicillin/sulbactam; TGC, tigecycline; VAN, vancomycin.

The CAST‐R results, via eMIC‐MA, showed an overall 93% CA (80 of 86 combinations) with BMD, yet achieving a 10‐fold reduction in TAT. Collectively, there are three major errors (ME), three minor errors (mE), and no very ME (VME; Tables 1 and S3). Specifically, CA is 96% for tigecycline, 90% for meropenem, 75% for ceftazidime, 87.5% for ampicillin/sulbactam, and 100% for vancomycin, oxacillin, clindamycin, and levofloxacin (Table 1). These results support the general applicability of CAST‐R.

## DISCUSSION

The deployment of phenotype‐based ASTs at clinical settings has been hindered by the inability to tackle slow‐growing pathogens (e.g., those via cellular morphology^24^ or weight^25^), or difficulty with specific and reliable measurement (e.g., those via electrochemical properties^26^). On the other hand, clinical application of the metabolism‐based Raman microspectroscopy‐enabled ASTs has faced the difficulty associated with the slow and tedious operation, which involves a large number of paralleled experimental procedures each consisting of sample pretreatment, cell wash, and Raman acquisition^14^, ^15^, ^27^. This is further exacerbated by the tandem nature of Raman microspectroscopy where the many drug‐exposure reactions spotted on a slide would need to be processed for Raman signal acquisition one by one (instead of all at once). Moreover, for last‐resort antibiotics such as tigecycline, whose sensitivity to the dissolved oxygen in broth has confounded proper interpretation of results from culture‐based AST, accuracy and reliability of the Raman‐based approaches have not been sufficiently validated on clinical samples.

In this study, a 3‐h, fully automated AST that tackles these challenges was demonstrated for tigecycline over a collection of clinical isolates and actual patient samples via the CAST‐R system. This represents several signs of progress. (i) High accuracy and reliability of eMIC‐MA: by incorporating MIL that normalizes against potential variation due to the change of strains, starting state of cells, or instruments, the metabolic‐activity‐based eMIC‐MA (notably, the metabolic state was recently shown to more accurately predict antibiotic lethality than growth rate^28^) produced quantitative AST results that are highly consistent with BMD (EA: 99%; CA: 93%). This is particularly important for tigecycline, as existing AST systems, such as VITEK 2 and E‐test, can often overestimate or even report false resistance^9^, ^10^. (ii) Automation of the manual steps: by developing a fully automated instrumentation solution (for plaque discrimination from sample chip, image auto‐focusing, and spectrum auto‐acquisition) and a neural‐network‐based filter (for intelligent spectrum‐quality assessment), CAST‐R reduces the full process from positive blood bottles to 3 h. This represents 10‐fold acceleration to BMD, which takes 36–48 h for tigecycline^7^. (iii) Broad applicability: as all cellular life forms alive would consume water, CAST‐R should be widely applicable to various prokaryotic and eukaryotic pathogens^11^, ^12^, ^13^, ^14^, ^15^, ^16^, and even to cancer cells^29^. Similarly, any antibiotics that inhibit vitality or abolish the viability of microbial cells can be tackled by this method.

Notably, the strengths of an SCRS, which also include information richness, single‐cell resolution, and ability to couple with downstream single‐cell sequencing^30^, have yet to be fully exploited in this study. These can be explored by additional technological development. For example, the CAST‐R workflow can be further accelerated by acquiring SCRS directly from infected body fluids (instead of the present requirement to wait for positive blood‐bottle results), by developing an efficient strategy for enriching the pathogen cells, or via flow‐mode Raman acquisition (e.g., the pDEP‐RADS‐enabled FlowRACS^31^). Moreover, as each SCRS can be pathogen‐specific^27^ and may reveal the mode‐of‐action of antimicrobials^32^ an integrated workflow extending from AST to pathogen identification and mechanism dissection should be possible. Finally, based on our recently introduced RAGE‐Seq^33^ or FlowRACS^31^, links between drug‐resistant phenotype and full genome sequence can be established at the precisely one‐cell resolution, to cross‐validate AST results and to track the emergence and spread of superbugs. Therefore, future efforts that integrate these features into CAST‐R should usher in new applications in basic and clinical investigations that combat superbugs.

## MATERIALS AND METHODS

### Strains and growth conditions

Clinical isolates of *A. baumannii* (*n* = 100) were collected from Peking Union Medical College Hospital, and each was identified by 16S recombinant DNA sequencing. Clinically, PBCs (*n* = 26) were collected from Qingdao University Affiliated Hospital (Qingdao). *E. coli* ATCC 25922, the recommended reference strain for AST, and *A. baumannii* ATCC 19606 were both purchased from American Type Culture Collection (ATCC; Manassas).

To build the database of high‐quality reference SCRS for properly and efficiently assessing SCRS quality, 13 species (15 strains) of the most frequently encountered pathogens in blood infections were collected (Table S1). For each of the strains, a single colony was picked from Columbia blood plate after overnight incubation, and then grown in CAMHB for 2 h at 37^◦^C, before SCRS acquisition.

For spiked PBCs, 14 human blood samples (9 ml each), each from a healthy subject, were respectively spiked with 1 ml suspension of the selected *A. baumannii* strains with a final concentration of 10^4^ CFU/ml as blood‐culture vials (BACTECTM Plus Aerobic/F culture vial; Becton Dickinson). Blood‐culture bottles were incubated at 37^◦^C in an automated blood‐culture chamber (BACTECTM FX400; Becton Dickinson) until flagged positive. The 14 selected *A. baumannii* strains for the blood culture test exhibit different susceptibility levels to tigecycline, with their MICs ranging from 0.125 to 8 mg/l (Table 1).

For the tests of eight antibiotics on 26 actual clinical blood cultures that reported positive (details provided in Table S3), the standard BMD method was employed as a control, according to the CLSI protocol^20^. For eMIC‐MA measurement via CAST‐R, the concentrations of each antibiotic tested were: 0.03–8 μg/ml for tigecycline (8 concentrations), 0.12–32 μg/ml for meropenem (9 concentrations), 0.25–2048 μg/ml for ceftazidime (11 concentrations), 2–512 μg/ml for ampicillin/sulbactam (5 concentrations), 0.12–1024 μg/ml for oxacillin (13 concentrations), 0.03–256 μg/ml for clindamycin (11 concentrations), and 0.25–4 μg/ml for vancomycin (6 concentrations) and levofloxacin (6 concentrations). An antibiotic‐free group was always enrolled as the control.

### Determination of MIC

MICs were determined via the BMD method using freshly prepared CAMHB in 96‐well plates. The colony suspension of each strain was adjusted to 0.5 Mcfarland standard before addition into wells, with the final concentration as 5 × 10^5^ CFU/ml. When performing AST for tigecycline, cells were incubated in freshly prepared (<6 h) cation‐adjusted Mueller‐Hinton broth (CAMHB) supplemented with 25 mg/l Ca^2+^ and 12.5 mg/l Mg^2+^ containing series doses of tigecycline.

For each test, tigecycline solution was freshly diluted by CAMHB from one aliquot of the stock solutions (16 g/l, prepared from tigecycline powder; Sigma‐Aldrich), which was stored at −80°C and covered with aluminum foil to avoid potential degradation. The tigecycline concentrations in plates spanned a doubling dilution range from 0.0625 to 64 mg/l. MIC is defined as the minimum concentration at which an antimicrobial can inhibit visible bacterial growth after 20 h incubation at 37°C in a non‐CO_2_ incubator as recommended by CLSI^20^. In the end, the tigecycline susceptibility results were interpreted according to EUCAST ECOFF (S: ≤ 0.5 mg/l; NS: ≥ 1 mg/l)^20^.

### Establishing D_2_O‐Ramanometry‐based AST workflow for tigecycline

To construct the D_2_O‐Ramanometry‐based AST pipeline, tigecycline‐S strain A29 and tigecycline‐NS strain A30 were harvested at the logarithmic growth phase, respectively, and cell density was adjusted to 5 × 10^5^ CFU/ml with MHB medium containing 0.125–8 mg/l tigecycline. Then 1 ml cells were plated in each well of a 96‐well plate, and cells were cultivated at 37°C for 1 h at 200 rpm. Afterwards, D_2_O was added to the culture medium to reach a final concentration of 30%, and the cells were incubated for another 1 h. Cells were collected and stored at 4°C before Raman spectrum acquisition. Each experiment was carried out in triplicate. To distinguish the two strains, the cutoff was determined according to the cross point of the normal distribution curve of MIL as reported^20^.

*A. baumannii* clinical isolates (*n* = 100) were used to validate the accuracy of CAST‐R based on the  pipeline. EA was defined as the eMIC‐MA from CAST‐R, that is, within ±1 fold dilution of the MIC from BMD. CA was calculated via correct susceptibility result from CAST‐R as compared to that from BMD. Errors were defined as^7^: (i) VME (CAST‐R = S and BMD method = NS), and (ii) ME (CAST‐R = NS and reference method = S).

### Automated multisample preparation for AST from PBC via CAST‐R

The PLS consists of three modules: incubation module, wash module, and spotting module. First, 1 ml of PBC was transferred into a 96‐well assay and co‐incubated with ACK lysis buffer (Beyotime) for 10 min. MHB medium, tigecycline, and D_2_O were added to each well automatically via eight‐channel pipettor according to the presetting procedure. Second, the samples were transferred to the vacuum‐based 96‐well cleaning module and washed with distilled water three times. The bacterial cells were suspended in 15 μl distilled water. Finally, 1 μl of cell suspension was transferred into a hydrophobic quartz slide, spotted in an array‐based pattern and subject to air drying.

### Automated acquisition and quality control of Raman spectra in CAST‐R

The spotted quartz slide was loaded into a CAST‐R (Qingdao Single‐Cell Biotechnology, Co., Ltd.). The CAST‐R carries a microscope with 100× dry objective (NA = 0.80; Leica) and a 532 nm Nd:YAG laser (Ventus; Laser Quantum Ltd.) with a maximum power of 50 mW. Each cell was exposed to a laser for 2 s and spectra were recorded with 300 grooves/mm diffraction grating.

Multiple‐sample locating was performed in two steps: precise positioning and auto‐focus. In addition, a discrimination method was designed for quick searching of targets to remove the randomness of plaque burst. Specifically, the position of a plaque was determined as a bright spot shown on the microimage. A Gaussian filter was then applied to reduce high‐frequency noises after converting the image into grayscale. By setting an optimized threshold, the plaque was located according to the brightness value, and the area of the dense bright spot was then chosen as the region of interest to be investigated.

During auto‐focusing and plaque boundary recognition, several image definition functions (e.g., the Tenengrad gradient function, etc.) were employed for image sharpness evaluation. This function retains gradient mutation points, ignores the gradient value of part of the blurred image, and only highlights the gradient energy value of the relatively clear image. These features enhance the efficiency of calculation and evaluation. In the focus search process, a curve fitting method was embedded in the local search of a traditional hill‐climbing algorithm, which improves the focused search strategy. For automatic recognition of plaque boundary, the canny algorithm was used to perform edge detection and contour extraction on cells, and the area with a large contour was selected for Raman spectrum collection.

Subsequently, CAST‐R automatically initiates the spectrum acquisition process and adaptively adjusts the detection parameters to obtain high‐quality Raman spectra. To properly and efficiently assess the quality of acquired Raman spectra, an intelligent filter based on CNN was developed. First, over 10,000 high‐quality spectra (SNR ≥ 30) from 13 species (15 strains) of pathogens that are most frequently encountered in blood infections were collected, so that a reference pathogen Raman database was created (Table S1). Then the CNN algorithm was employed to compare the acquired spectra with those in the reference database^27^. Specifically, the Raman spectra to be evaluated were employed as network input; after calculation at the convolution layer, the pooling layer, and the full connection layer, an *n*‐dimensional output vector corresponding to the *N* species in the reference Raman database (*N* = 13 at present) was produced; then this vector was mapped to the softmax function^34^ as the input, to obtain the probabilities of correct assignment to each species. If the maximal probability of an input Raman spectrum is higher than the probability threshold designated, the quality of the input spectrum is considered as high.

In our experiments, at least 100 Raman spectra that pass the quality control above were acquired in each sample. All spectra underwent background noise subtraction, baseline correction, and normalization using the Q‐specs software (Qingdao Single‐Cell Biotechnology, Co., Ltd.). C–D ratio was calculated by dividing the C–D band (2040–2300 cm^−1^) to the sum of the C–D band and C–H band (2800–3100 cm^−1^).

### Statistical analysis

Statistical analysis of the C–D ratio and MIL was performed using R (Version 4.0). Graphics were produced via the ggplot 2 package.

## AUTHOR CONTRIBUTIONS

Pengfei Zhu, Jian Xu, and Qiwen Yang conceived the project. Jing Dai, Huijie Liu, Yuli Mao, Ying Zhu, Lili Zhang, and Xiaoting Fu performed MIC and eMIC‐MA measurements. Pengfei Zhu, Jian Xu, Lihui Ren, Rongze Chen, and Lijun Sun constructed the reference pathogen Raman spectra database, developed CAST‐R experimental workflow, and analyzed data. Bo Ma, Yuandong Li, Lihui Ren, Xiaoshan Zheng, and Jian Xu developed a CAST‐R instrument and associated chips for the acquisition of pathogen Raman spectra. Ying Zhu, Yingchun Xu, Yuanqi Zhu, and Qiwen Yang provided clinical samples. Jian Xu, Pengfei Zhu, Lihui Ren, Jing Dai, and Qiwen Yang wrote the paper.

## ETHICS STATEMENT

Protocols of this study were reviewed by the Human Research Ethics Committee of the Institutional Review Board (IRB) of the Peking Union Medical College Hospital. As all bacterial strains were from residual samples used in clinical diagnosis or strains from their subcultures, the criteria for exemption were met. Moreover, as this project did not affect the normal diagnosis and treatment of patients, after consultation with the IRB, formal ethical approval was reviewed and waived, and written patient consent was not required (Ethics Approval Number: HS‐2914).

## CONFLICT OF INTERESTS

Professors Jian Xu and Bo Ma are among the founders of Qingdao Single‐Cell Biotechnology Co., Ltd.

## Supporting information

Supporting information.

Supporting information.

Supporting information.

Supporting information.

## Data Availability

The data that support the findings of this study are openly available at http://cast.single-cell.cn/.
