# Supplementary figures and images for: Rapid, automated, and reliable antimicrobial susceptibility test from positive blood culture by CAST‐R
Source: mLife. 2022 Apr 18;1(3):329–40. doi: 10.1002/mlf2.12019 (PMC10989881; doi:10.1002/mlf2.12019)

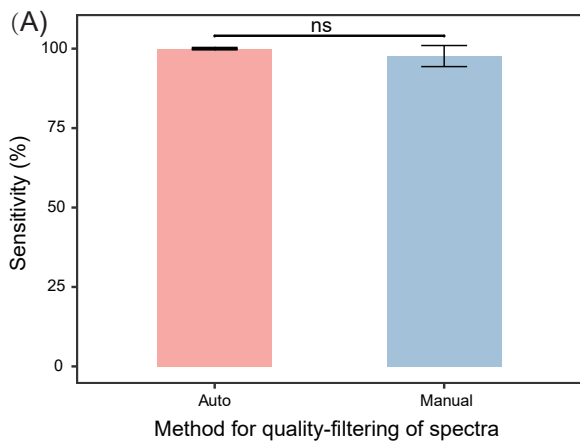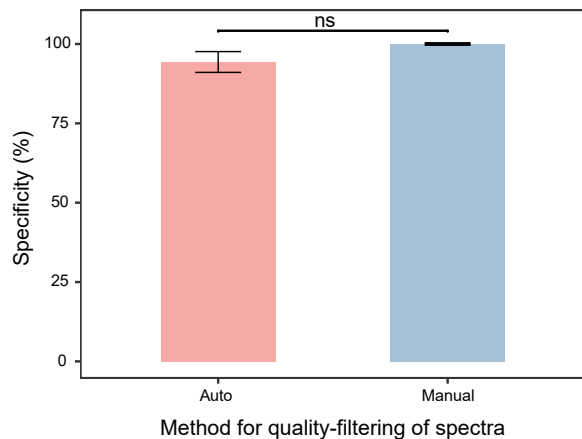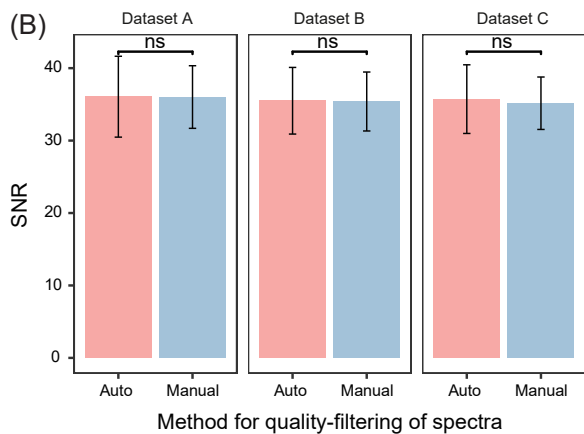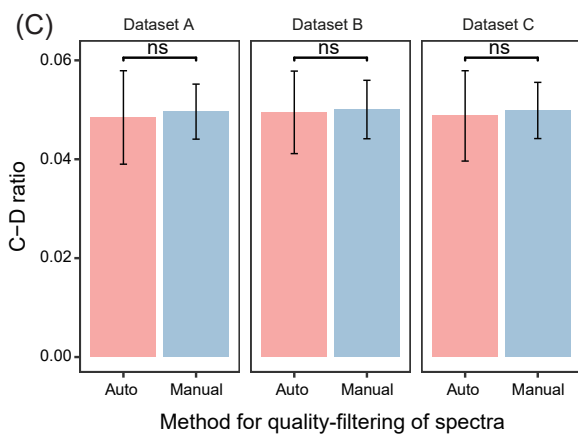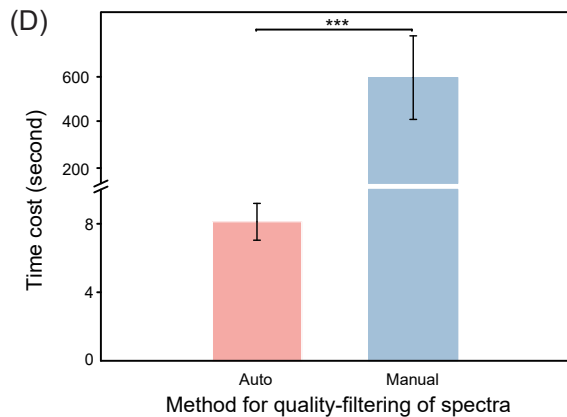

Supplement: Supplementary file 1 — Supporting information. [file MLF2-1-329-s004.pdf]

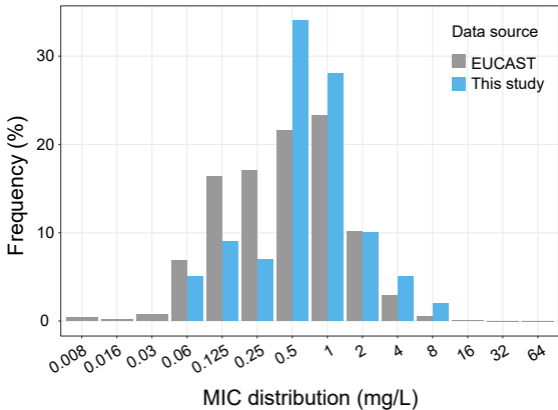

Supplement: Supplementary file 2 — Supporting information. [file MLF2-1-329-s001.pdf]

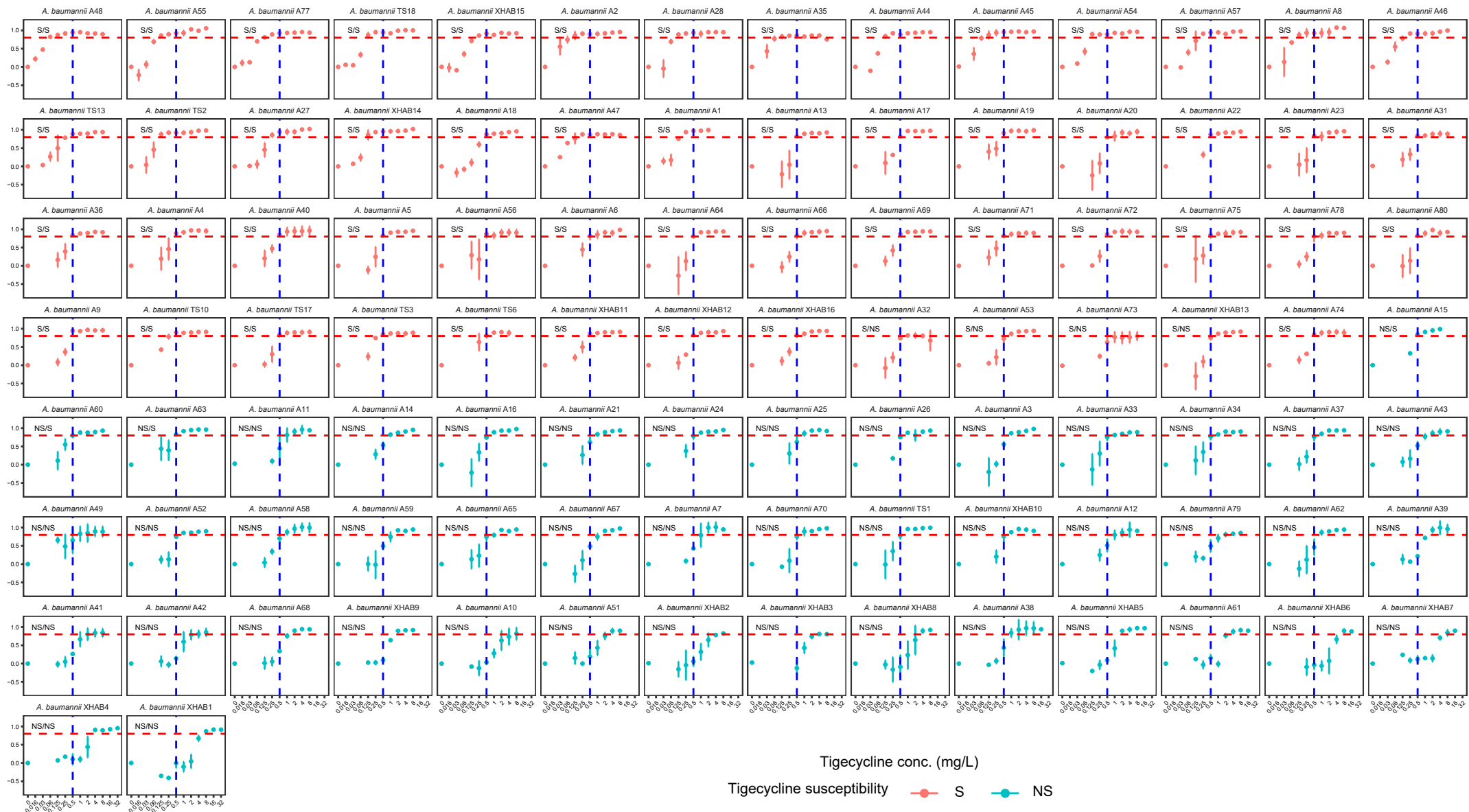

Supplement: Supplementary file 3 — Supporting information. [file MLF2-1-329-s002.pdf]
