# Supplementary material for: Rapid, automated, and reliable antimicrobial susceptibility test from positive blood culture by CAST‐R
Source: mLife. 2022 Apr 18;1(3):329–40. doi: 10.1002/mlf2.12019 (PMC10989881; doi:10.1002/mlf2.12019)
Supplement: Supplementary file 4 — Supporting information. [file MLF2-1-329-s003.docx]

**Rapid, automated and reliable antimicrobial susceptibility test from positive blood culture by CAST-R**

Pengfei Zhu^1,2,#^, Lihui Ren^1,2,3,#^, Ying Zhu^4,5,#^, Jing Dai^1,2,#^, Huijie Liu^1,2^, Yuli Mao^1,2^, Yuandong Li^1,2^, Yuehui He^1,2^, Xiaoshan Zheng^1,2^, Rongze Chen^1,2^, Xiaoting Fu^1,2^, Lili Zhang^1,2^, Lijun Sun^1,2^, Yuanqi Zhu^6^, Yuetong Ji^1,7^, Bo Ma^1,2^, Yingchun Xu^4^, Jian Xu^1,2,8^*, Qiwen Yang^4,^*

^1^Single-Cell Center, CAS Key Laboratory of Biofuels, Shandong Key Laboratory of Energy Genetics and Shandong Energy Institute, Qingdao Institute of Bioenergy and Bioprocess Technology, Chinese Academy of Sciences, Qingdao, Shandong, China

^2^University of Chinese Academy of Sciences, Beijing, China

^3^College of Information Science & Engineering, Ocean University of China, Qingdao, Shandong, China

^4^Department of Clinical Laboratory, Peking Union Medical College Hospital, Peking Union Medical College, Chinese Academy of Medical Sciences, Beijing, China

^5^Graduate School, Peking Union Medical College, Chinese Academy of Medical Sciences, Beijing, China

^6^Department of Clinical Laboratory, Affiliated Hospital of Qingdao University, Qingdao University, Qingdao, Shandong, China

^7^Qingdao Single-Cell Biotechnology, Co., Ltd., Qingdao, Shandong, China

^8^The Bioland Laboratory, Guangzhou, Guangdong, China

^#^These authors contributed equally to this work.

*Correspondence: Qiwen Yang, yangqiwen@pumch.cn, Jian Xu, xujian@qibebt.ac.cn

**Supplemental Tables and Figures**

**Table S1. A reference pathogen Raman spectra database for development of the intelligent spectra-quality filter in CAST-R.** The database consists of 13 species (15 strains) of the most common pathogens in blood infections.

| Species Name | Strain ID | | Num. of Raman Spectra | |
| --- | --- | --- | --- | --- |
| *Escherichia coli* | ATCC 25922 | 696 | |  |
| *Escherichia coli* | ATCC 35218 | 640 | |  |
| *Acinetobacter baumannii* | ATCC 19606 | 705 | |  |
| *Pseudomonas aeruginosa* | ATCC 27853 | 537 | |  |
| *Enterobacter cloacae* | ATCC 700323 | 862 | |  |
| *Klebsiella pneumoniae* | ATCC 700603 | 717 | |  |
| *Enterococcus faecalis* | ATCC 29212 | 983 | |  |
| *Enterococcus faecium* | ATCC 19434 | 647 | |  |
| *Enterococcus casseliflavus* | ATCC 700327 | 919 | |  |
| *Staphylococcus aureus* | ATCC 29213 | 642 | |  |
| *Staphylococcus aureus* | ATCC 25923 | 774 | |  |
| *Staphylococcus epidermidis* | ATCC 12228 | 550 | |  |
| *Staphylococcus sciuri* | ATCC 29061 | 414 | |  |
| *Staphylococcus hominis* | ATCC 27844 | 495 | |  |
| *Staphylococcus capitis* | ATCC 49326 | 319 | |  |

**Table S2. MICs and eMIC-MAs of spiked positive blood cultures**. The antimicrobial susceptibility results measured via eMIC-MA are consistent with those based on MICs.

| **Positive blood culture** | **Pathogen** | **MIC by BMD (mg/L)** | **tigecycline susceptibility via MIC** | **eMIC-MA (mg/L)** | **tigecycline susceptibility via eMIC-MA** |
| --- | --- | --- | --- | --- | --- |
| PBC-1 | *A. baumannii* | 1 | NS | 1 | NS |
| PBC-2 | *A. baumannii* | 0.125 | S | 0.125 | S |
| PBC-3 | *A. baumannii* | 0.25 | S | 0.25 | S |
| PBC-4 | *A. baumannii* | 4 | NS | 4 | NS |
| PBC-5 | *A. baumannii* | 8 | NS | 4 | NS |
| PBC-6 | *A. baumannii* | 0.5 | S | 0.5 | S |
| PBC-7 | *A. baumannii* | 1 | NS | 1 | NS |
| PBC-8 | *A. baumannii* | 0.25 | S | 0.25 | S |
| PBC-9 | *A. baumannii* | 2 | NS | 2 | NS |
| PBC-10 | *A. baumannii* | 0.5 | S | 0.5 | S |
| PBC-11 | *A. baumannii* | 2 | NS | 0.5 | S |
| PBC-12 | *A. baumannii* | 0.125 | S | 0.25 | S |
| PBC-13 | *A. baumannii* | 8 | NS | 16 | NS |
| PBC-14 | *A. baumannii* | 4 | NS | 4 | NS |

**Table S3. MICs and eMIC-MAs of the eight antibiotics on 26 actual clinical blood cultures that reported positive.** The antimicrobial susceptibility results measured based on eMIC-MA (via the CAST-R system) are consistent with those based on MICs (via the BMD method), with categorical agreement of 93%. TGC: tigecycline; MEM: meropenem; CAZ: ceftazidime; SAM: ampicillin/sulbactam; OXA: oxacillin; CLI: clindamycin; VAN: vancomycin; LVX: levofloxacin. Four of the most frequently encountered pathogens in hospitals in China were included: *Escherichia coli*, *Klebsiella pneumonia*, *Staphylococcus aureus* and *A. baumannii*. Discrepant AST results are marked with underlines. Full experimental details are provided in **Supplemental Methods**.

| **Positive blood culture** | **Pathogen** | **MIC/eMIC-MA (μg/mL)** | | | | | | | |
| --- | --- | --- | --- | --- | --- | --- | --- | --- | --- |
|  |  | **TGC** | **MEM** | **CAZ** | **SAM** | **OXA** | **CLI** | **VAN** | **LVX** |
| Abau-1 | *A. baumannii* | 2/2 | 64/>32 | >256/32 | 64/32 | - | - | - | - |
| Abau-2 | *A. baumannii* | 4/4 | >64/>32 | - | - | - | - | - | - |
| Abau-3 | *A. baumannii* | 2/2 | 64/>32 | - | - | - | - | - | - |
| Abau-4 | *A. baumannii* | 8/2 | 64/>32 | - | - | - | - | - | - |
| Abau-5 | *A. baumannii* | 0.25/0.5 | 0.25/4 | - | - | - | - | - | - |
| Abau-6 | *A. baumannii* | 4/4 | 0.25/4 | - | - | - | - | - | - |
| Abau-7 | *A. baumannii* | 0.5/1 | 8/>32 | 8/512 | 8/8 | - | - | - | - |
| Abau-8 | *A. baumannii* | 2/1 | >64/>32 | - | - | - | - | - | - |
| Abau-9 | *A. baumannii* | 2/2 | >64/>32 | - | - | - | - | - | - |
| Eco-1 | *E. coli* | 0.125/<0.5 | 0.06/<0.25 | 64/2048 | 64/64 | - | - | - | - |
| Eco-2 | *E. coli* | 0.25/<0.5 | 0.06/<0.5 | - | - | - | - | - | - |
| Eco-3 | *E. coli* | 0.06/<0.5 | <0.12/<1 | - | - | - | - | - | - |
| Eco-4 | *E. coli* | 0.25/<0.5 | <0.12/0.5 | 4/16 | 16/32 | - | - | - | - |
| Eco-5 | *E. coli* | 0.125/<0.5 | <0.12/0.5 | <0.25/1 | 2/4 | - | - | - | - |
| Eco-7 | *E. coli* | 0.125/<0.5 | 0.06/0.5 | - | - | - | - | - | - |
| Eco-8 | *E. coli* | 0.125/<0.5 | 0.06/0.5 | - | - | - | - | - | - |
| Kpn-1 | *K. pneumoniae* | 0.25/<0.5 | <0.12/4 | <0.25/4 | 2/4 | - | - | - | - |
| Kpn-2 | *K. pneumoniae* | 0.25/0.5 | <0.12/0.5 | - | - | - | - | - | - |
| Kpn-3 | *K. pneumoniae* | 0.25/0.5 | <0.12/1 | 4/4 | >64/64 | - | - | - | - |
| Kpn-4 | *K. pneumoniae* | 0.25/0.5 | <0.12/2 | <0.25/0.25 | 2/2 | - | - | - | - |
| Sau-1 | *S. aureus* | 0.125 | - | - | - | 16/32 | >64/>8 | 0.5/1 | 0.5/0.25 |
| Sau-3 | *S. aureus* | 0.125 | - | - | - | 1/1 | 0.12/<0.12 | 0.5/1 | 0.5/1 |
| Sau-5 | *S. aureus* | 0.125 | - | - | - | 0.5/2 | 0.12/0.12 | 1/1 | 0.12/0.25 |
| Sau-6 | *S. aureus* | 0.125 | - | - | - | 0.25/1 | 0.12/0.25 | 0.5/1 | 0.5/1 |
| Sau-7 | *S. aureus* | 0.125 | - | - | - | 64/128 | 0.12/0.12 | 1/1 | 2/1 |
| Sau-8 | *S. aureus* | 0.25 | - | - | - | >64/32 | >64/>8 | 1/1 | 0.5/0.25 |

**Figure S1. Intelligent filter in CAST-R for automated quality assessment and screening of Raman spectra.** Advantages of the machine-learning filter was apparent, based on comparison with the manual operation (via expert users), for four performance parameters including the proportion of high-quality spectra (**A**), SNR (**B**), C-D ratio (**C**) and time cost (**D**) (Wilcox test; ***, *p*≤0.001; ns, *p*>0.05). Each dataset contains 100 spectra.

**Figure S2. Comparison of MIC distribution patterns for tigecycline between the** ***A. baumannii* isolates in EUCAST and those selected for validating CAST-R performance in this study.** The data collection for tigecycline susceptibility of *A. baumannii* from EUCAST (31114 observations) was used as the reference for guiding the selection of isolates for validating CAST-R performance. One hundred isolates were enrolled in this study. The MIC of each of these isolates was measured via the BMD method.

**Figure S3. Rapid tigecycline AST of clinical *A. baumannii* isolates via CAST-R.** The distribution of Metabolic Inhibition Level of *A. baumannii* (n=100) at the tigecycline concentration of 0~32 mg/L respectively. The blue vertical dashed lines are the breakpoint of MIC to determine susceptibility (0.5 mg/L), while the red horizontal dashed lines (0.8) are the criteria to determine eMIC-MA (S, mean MIL_0.5 mg/L_ <0.8; NS, mean MIL_0.5 mg/L_ ≥0.8). At the top left corner of each panel, the tigecycline susceptibility results via BMD (left) and via CAST-R (right) are provided respectively. S, susceptible strain; NS, Non- susceptible strain.
